# Decabromodifenyl Ether (BDE-209) in Surface Soils from Warsaw and Surrounding Areas: Characterization of Non-Carcinogenic Risk Associated with Oral and Dermal Exposure

**DOI:** 10.3390/molecules29102335

**Published:** 2024-05-16

**Authors:** Wojciech Korcz, Katarzyna Czaja, Monika Liszewska, Radosław Lewiński, Anna Słomczyńska, Paweł Struciński

**Affiliations:** Department of Toxicology and Health Risk Assessment, National Institute of Public Health NIH—National Research Institute, Chocimska 24, 00-791 Warsaw, Poland

**Keywords:** polybrominated diphenyl ethers, surface soil, oral and dermal intake, non-carcinogenic health risk

## Abstract

Polybrominated diphenyl ethers (PBDEs) have been used for many years as flame retardants. Due to their physicochemical and toxicological properties, they are considered to be persistent organic pollutants (POPs). BDE-209 is the main component of deca-BDE, the one PBDE commercial mixture currently approved for use in the European Union. The aim of this study was to analyse BDE-209 in surface soil samples from Warsaw and surrounding areas (Poland) as an indicator of environmental pollution with PBDEs, and to characterise the associated health risk. A total of 40 samples were analysed using gas chromatography with electron capture detection (GC-µECD). Concentrations of BDE-209 in soil ranged from 0.4 ng g^−1^ d.w. (limit of quantification) to 158 ng g^−1^ d.w. Overall, 52.5% of results were above the method’s limit of quantification. The highest levels were found at several locations with heavy traffic and in the vicinity of a CHP plant in the city. The lowest concentrations were observed in most of the samples collected from low industrialized or green areas (<0.4 to 1.68 ng g^−1^ d.w.). Exposure to BDE-209 was estimated for one of the most sensitive populations, i.e., young children. The following exposure routes were selected: oral and dermal. No risk was found to young children’s health.

## 1. Introduction

Polybrominated diphenyl ethers (PBDEs) have been used for many years as flame retardants. They were added to many consumer products such as textiles, furniture, plastic products, and electronic equipment. PBDEs are highly persistent in the environment. They can bioconcentrate in animal and human tissue as well as being able to biomagnify in food webs. Furthermore, due to their ability to be transported over long distances, e.g., in dust, they are widespread in the environment [1,2,3]. Therefore, these compounds are considered persistent organic pollutants (POPs) [4].

The main sources of emissions of these compounds are equipment containing PBDEs (e.g., plastics, electronics and textiles) and landfills (containing electronic and electrical waste and plastic waste), from which these compounds are released into the environment, among other sources, as a result of photodegradation, abrasion, incineration and waste treatment, and leakage into surface and groundwater [3,4,5,6,7]. The use of municipal sewage sludge containing PBDEs as fertilizer can also lead to additional environment pollution [8,9]. Additionally, the increasing prevalence of plastics in the marine environment impacts the problem of pollution [10,11].

The use of PBDEs has been restricted by international regulations such as Directive 2003/11/EC on the restriction of the use of hazardous substances in the European Union [4,12,13] and the Stockholm Convention on Persistent Organic Pollutants (POPs) [14,15,16]. Despite these limitations, these compounds are still detected in samples from various elements of the environment and in food [17]. Plants can absorb PBDEs from soil and water, and thus cause food contamination [18,19,20]. The deca-BDE is currently the only flame-retardant PBDE commercial mixture approved for use in the European Union. It consists mainly (up to 97%) of the decabromodiphenyl ether (BDE-209) [21]. BDE-209 is also the dominant congener found in soil samples [17,22].

The main routes of human exposure to PBDEs are oral intake via food and dust, inhalation, and dermal absorption. Exposure resulting from the intake of these compounds by inhalation and dermal absorption is much lower than that from ingestion. 

PBDEs intake may pose a risk to human health. In animal studies, many adverse effects were observed. It has been shown that these compounds disrupt the hormonal balance of mammals and have neurotoxic effects. They may cause reproductive disorders in vertebrates. Furthermore, they cause damage related to oxidative stress in various organs [17,23]. The aim of this study was to analyse BDE-209 congener in soil samples from Warsaw and surrounding areas (Poland) as an indicator of environmental pollution with polybrominated diphenyl ethers. The next goal was to characterise the health risks associated with dermal and oral exposure.

## 2. Results and Discussion

### 2.1. Levels of BDE-209 in Surface Soils

Concentrations of BDE-209 in soil obtained in this study from Warsaw and the surrounding area ranged from 0.4 ng g^−1^ d.w. (limit of quantification, LOQ) to 158 ng g^−1^ d.w. In total, 52.5% were above the LOQ. The mean was 8.9 ng g^−1^ d.w., and the median was 1.0 ng g^−1^ d.w. (Table 1).

Furthermore, the median was lower than the mean, which is typical for right-skewed distribution [24]. Results found in this study were lower than those analysed in earlier studies of indoor dust [25,26]. In addition, widespread BDE-209 occurrence has also been observed, e.g., in samples taken from less urbanised areas such as forests near Warsaw. This supports the selection of BDE-209 as an indicator of environmental pollution with polybrominated diphenyl ethers. 

Levels of decabromodiphenyl ether found in this study were characterised by high dispersion. The same spread of results was also observed by other authors (Table 2). 

Similar levels of BDE-209 in soil samples from urbanised areas in Korea were determined by Jeon et al. [35]. In contrast, results found by Dreyer et al. [33] in samples from Germany were an order of magnitude lower. Surface soils taken from the vicinity of landfills including e-waste, as well as from the vicinity of plastic factories and recycling sites (from different locations around the world), contained much higher levels of decabromodiphenyl ether [27,28,31,32,34,36]. Based on this, it seems reasonable to claim that these sites may be major sources of environmental contamination. Soil from less urbanised and rural sites was less contaminated with this PBDE congener [31,35,36]. Likewise, in this study, the lowest concentrations were observed in most of samples collected from low industrialised or green areas (<0.4 to 1.68 ng g^−1^ d.w.). In the case of decabromodiphenyl ether, the predominant mechanism of its “transfer” into the environment (from materials containing this compound) is through abrasion, in which particles of material containing decabromodiphenyl ether are ”released” and carried by air or by water (run-off and leaking) [37,38]. Zhang et al. showed a significant negative correlation of BDE-209 concentrations with the distance from an electro-waste disposal facility [22]. Therefore, soils from suburban and agricultural areas located far from potential sources of BDE-209’s release into the environment is less contaminated. In the present study, the highest levels were found at several locations with heavy traffic and in the vicinity of a CHP plant in the city (up to the maximum of 158 ng g^−1^ d.w.).

### 2.2. Non Carcinogenic Human Health Risk Characterization

The exposure of small children to decabromodiphenyl ether ingested through soil and by dermal absorption was estimated. Low (P50) and high (P95) exposure scenarios were considered. Furthermore, non-carcinogenic human health risk was characterised. The results are presented in Table 3. 

Under both exposure scenarios, health quotients (HQs) were low. Therefore, risks resulted from BDE-209 intake via dermal and oral exposure routes can be considered negligible. The highest exposure result corresponded to 1.5% RfD. Similar results were obtained by other authors in the case of soil from both agricultural [36,39] and urbanised areas, as well as from landfills or e-waste recycling sites [5,31,36,40]. 

It should be noted that some studies’ results cannot be fully compared due to different assumptions used in calculations, such as different bioavailability factors or dermal absorption coefficients. In our study, only one PBDE congener was analysed (dominant congener in soil). Despite this, after the literature review, conclusions from the reviewed studies were consistent and indicated a low health risk. Authors who tested the sum of selected congeners also found no risk [5,31,40]. However, soil pollution by PBDEs should be controlled because these compounds are constantly released into the environment. 

## 3. Material and Methods

### 3.1. Sample Collection

Forty samples of surface soils from Warsaw and its suburbs were collected between 2019 and 2022. They were collected, among others, from places with heavy traffic, the vicinity of industrial and waste processing plants, in municipal parks and forests. Samples were dried and sifted through a 500 µm steel sieve using Retsch AS 200 basic shaker (Retsch GmbH, Haan, Germany) and stored in aluminium bags until analysis at −20 °C. 

### 3.2. Standard, Reagents, and Chemicals

BDE-209 standard, in the form of a 1.2 mL ampule with a 50 µg mL^−1^ concentration in nonane was purchased from Cambridge Isotope Laboratories (Andover, MA, USA). ECD and FID gas chromatography grade *n*-hexane and acetone were acquired from Merck (Darmstadt, Germany). Dichloromethane for the analysis of pesticides residue, *n*-dodecane for synthesis, silica gel 60 extra pure (70–230 mesh ASTM) (Merck KGaA, Darmstadt, Germany) for column chromatography, glass wool, and aluminium oxide 90 active, neutral for column chromatography were also obtained from Merck (Darmstadt, Germany). Sodium sulphate anhydrous (12–60 mesh) was provided by J.T. Baker (Deventer, The Netherlands), and sea sand extra pure was provided by Supelco (Darmstadt, Germany). Cellulose filter tubes (43 × 123 mm) were acquired from Munktell (Barestein, Germany).

### 3.3. Analytical Method 

An amount of 25 g of soil was extracted and purified using a slightly modified method (without florisil addition during the extraction step), described by Korcz et al. [26,41]. In brief, the extraction was made with a 3:1 (*v*/*v*) mixture of n-hexane–acetone using an automated Büchi B-811extraction system. Clean-up procedure was performed in a glass column (60 cm × 1.5 cm i.d.) containing the following layers, from the bottom: 0.5 cm of sodium sulphate anhydrous, 10 g of silica gel, 5 g of aluminium oxide and 0.5 cm of sodium sulphate anhydrous. Then, the samples were analysed by gas chromatography with electron capture detection (GC-µECD). Information on chromatographic settings used for BDE-209 quantification is presented in Table 4.

Confirmation of identity was performed using another GC-µECD method with a different chromatographic column. Details of the method are presented in Table 5.

### 3.4. Results of Validation of the Analytical Method Used for Analysing BDE-209

The analytical method’s recovery and precision were tested using the sea sand samples spiked with BDE-209 at 3 levels of fortification (I-0.4 ng g^−1^ d.w., II-4 ng g^−1^ d.w. and III-20 ng g^−1^ d.w.). Six samples per level were analysed. Recovery ranged from 71% to 130%, and mean recovery was 104%, 89% and 96%, respectively. Relative standard deviations (RSD%) calculated at these levels were 15% (I), 13% (II) and 16% (III). A calibration curve was determined using seven standard solution concentrations. A coefficient of determination (R^2^) ≥ 0.999 was obtained. The LOQ (limit of quantification) was set at 0.4 ng g^−1^ d.w. All the results were reported within the validated working range (0.4–20 ng g^−1^ d.w.). 

The identity of BDE-209 was confirmed using another analytical method, whose working range corresponded to the analogous range of the primary method. The linearity and limit of quantification of this method were acceptable.

Based on the validation parameters, the analytical method is acceptable for analysing BDE-209 in tested samples.

### 3.5. BDE-209 Levels, Exposure Estimation, and Non-Carcinogenic Human Risk Characterization 

For statistical purposes, when results were below the limit of quantification, an LOQ value was assigned accordingly to the upper bound approach [42].

This study results at low (median) and high (P95) BDE-209 concentration in soil were used for exposure calculations. Young children, as one of the most vulnerable populations, were selected. Furthermore, in case of exposure, one of the most sensitive human sub-age populations, i.e., young children was considered for exposure estimation. 

The exposure to BDE-209 congener ingested with soil was calculated using the following Equation (1) [26]:

(1) $$E_{ing}=C\times \frac{\;IR\times EF}{BW},$$

where E_ing_—exposure via ingestion, C—concentration of BDE-209, IR—daily intake rate, EF—exposure frequency factor, and BW—body weight. 

The U.S. Environment Protection Agency (US EPA) recommends values for daily soil ingestion [43] be used to estimate the IR parameter. In the case of young children (2–6 years), recommended values were 30 mg day^−1^ (general population central tendency—the value used for the low exposure scenario), and 90 mg day^−1^ (general population upper percentile—value used for the high exposure scenario). According to EFSA recommendations, a body weight of 12 kg was adopted for young children [44]. Six hours of outdoor activity per day ($\frac{1}{4}$) was used for the EF parameter.

In addition, dermal exposure to BDE-209 was estimated using the following Equation (2) [45]:

(2) $$E_{der}=C\times \frac{SA\times AF\times ABS\times EF\times ED}{BW\times AT}\times CF,$$

where E_der_—exposure via dermal contact, C—concentration of BDE-209, SA—skin surface area, AF—skin adherence factor, ABS—dermal absorption factor, EF—exposure frequency factor, ED—exposure duration, BW—body weight, AT—average time, and CF—conversion factor.

The following values were used to estimate dermal exposure in the selected population. Skin surface areas (for children aged 2 to 3 years old) for the low-exposure and high-exposure scenarios were, respectively, 6100 and 7000 cm^2^ [46]. AF was adopted at 0.2 mg cm^−2^ per day [46], and a value of 0.1 was used for ABS [45]. ED was set at 4 years, and EF was 45 days per year. In the case of BW, 10 kg was considered [47]. AT (ED x 365 days) was 1460 days, and the CF value was 10^−6^ [45].

The Equation (3) was used to calculate hazard quotients (HQs) [48]:

(3) $$HQ=\frac{E}{RfD}$$

where calculated exposure values (E—E_ing_ or E_der_) were compared to the corresponding reference dose (RfD) value established by the U.S. Environment Protection Agency. The RfD value for BDE-209 was 0.007 mg kg^−1^ of b.w. day^−1^ [49].

When the calculated HQ is higher than or equal to 1, it is assumed that there is a potential non-carcinogenic risk of an adverse health outcome [50].

### 3.6. Literature Review

The following databases were included: Medline, Agricola, GreenFile and Academic Search Ultimate, eBook Collection (EBSCOhost). Furthermore, keywords such as PBDEs, BDE-209, decabromodiphenyl ether, decaBDE, soil, urban areas, PBDE + soil degradation, exposure, and risk were also used.

## 4. Conclusions

The presence of BDE-209 in the surface layer of soil from Warsaw and the surrounding area confirms the ubiquitous environmental pollution with polybrominated diphenyl ethers. Similarly to other matrices (e.g., household or office dust), our results are characterised by high spread, which is consistent with results of BDE-209 levels in soil in other countries and continents. It cannot be ruled out that the tested analyte may enter the human body through oral and dermal routes. Using a conservative exposure scenario, we presented that non-carcinogenic risk to the health of one of the most vulnerable subpopulations—young children—associated with the intake of BDE-209 present in soil via the alimentary route and by dermal exposure can be considered negligible. However, since deca-BDE is a persistent organic pollutants (POP), its levels in the environment should be constantly monitored.

## Figures and Tables

**Table 1:** Statistical parameters of the analytical results of BDE-209 concentrations in soil samples from Warsaw and the surrounding areas.

Statistics	BDE-209
Median	1.0 [ng g^−1^ d.w.]
Mean	8.9 [ng g^−1^ d.w.]
First quartile	0.4 [ng g^−1^ d.w.]
Third quartile	5.7 [ng g^−1^ d.w.]
95th percentile	29.3 [ng g^−1^ d.w.]
Skewness	5
Kurtosis	31

**Table 2:** Comparison of BDE-209 levels found in surface soils obtained in studies from different regions of the world.

Country	Details on Sampling Site	*N*	Mean	Median	Min	Max	Reference
			[ng g^−1^ d.w.]				
Australia	The vicinity of an e-waste recycling facility	18	6000	160	<17	98,000	[27]
	Inner-city urban parkland	8	19	20	ND *	43	[27]
Brazil	Landfill	7	110	4.9	ND	715	[28]
China	Steel production site	7	13.1	-	1.59	64.3	[29]
	Agriculture area	6	1.4	-	0.505	3.97	[29]
	Downstream of Chuhe river	15	-	0.32	ND	41.4	[30]
	Neighbourhood of plastic production manufacture	30	42.8	-	2.21	400	[31]
	Neighbourhood of e-waste recycling facility	4	1500	1440	30	3100	[32]
	The vicinity of an abandoned E-waste dismantling facility	253	25.39	19.77	ND	857.72	[22]
Germany	Leipzig conurbation	4	0.69	-	ND	1.57	[33]
Ghana	e-waste processing and dumping site	15	1600	-	ND	8800	[34]
Korea	Suburban sites	24	2.52	-	0.6	10.6	[35]
	Urban sites	24	10	-	0.6	94.9	[35]
	Industrial sites	13	916	-	0.6	11,318	[35]
Sierra Leone	General waste site	10	23	-	ND	87	[34]
Taiwan	Neighbourhood of plastic production manufacture	15	1772	604	130	5430	[36]
Poland	Warsaw and surroundings	40	8.9	1.0	<0.4	158	This study

* ND—not detected.

**Table 3:** Assessment of long-term exposure to BDE-209 in young children following oral and dermal intake with soil. Characterization of associated risks (HQs).

Results	
Oral intake (low exposure scenario)	6.25 × 10^−10^ mg kg^−1^ b.w.
Oral intake (high exposure scenario)	5.5 × 10^−8^ mg kg^−1^ b.w.
Dermal intake (low exposure scenario)	3.2 × 10^−6^ mg kg^−1^ b.w.
Dermal intake (high exposure scenario)	1.1 × 10^−4^ mg kg^−1^ b.w.
HQ (oral; low exposure scenario)	1 × 10^−7^
HQ (oral; high exposure scenario)	7.9 × 10^−6^
HQ (dermal; low exposure scenario)	4.6 × 10^−4^
HQ (dermal; high exposure scenario)	1.5 × 10^−2^

**Table 4:** The chromatographic settings (Agilent Technologies 6890N) (Agilent, Wilmington, NC, USA) used for quantification of the BDE-209 congener.

Settings ^1^	
Column	DB-XLB (15 m × 0.25 mm i.d. and film thickness 0.1 µm)
Oven temperature program	120 °C (1 min)–30 °C min^−1^–300 °C (8 min)
Flow mode	Ramp flow
Carrier gas flow ramp program	1.5 mL min^−1^ (7 min)–15 mL min^−1^–3 mL min^−1^
PTV injector	“solvent vent mode”, ramp temperature program: 40 °C (0.3 min)–700 °C min^−1^–285 °C (3 min)
Injection volume	1 μL
Carrier gas	helium
Makeup (nitrogen)	30 mL min^−1^
Detector temperature	335 °C

^1^ The retention time for BDE-209 is 13.55 min.

**Table 5:** The chromatographic settings (Agilent Technologies 6890N) used for confirmatory purpose.

Settings ^1^	
Column	Rtx-1614 (15 m × 0.25 mm i.d. and film thickness 0.1 µm)
Oven temperature program	120 °C (1.5 min)–15 °C min^−1^–300 °C–5 °C min^−1^–310 °C (4 min)
Flow mode	Constant flow
Carrier gas flow	2.2 mL min^−1^
PTV injector	“solvent vent mode”, ramp temperature program: 40 °C (1.5 min)–700 °C min^−1^–400 °C (3 min)
Injection volume	3 × 1 μL (delay 10 s)
Carrier gas	helium
Makeup (nitrogen)	30 mL min^−1^
Detector temperature	345 °C

^1^ The retention time for BDE-209 is 13.70 min.

## Data Availability

Data are contained within the article.
